# 

**Published:** 1879-02

**Authors:** 


### BOOKS AND PAMPHLETS RECEIVED.

The Prophylaxis of Puerperal Convulsions. By E. S. Dunster, M.D. Reprint from Toledo *Medical and Surgical Journal.*

Fourth Annual Report of the Officers and Superintendent of the Asylum at Walnut Hill, Hartford, Connecticut, at their Annual Meeting, Oct. 9, 1875. Also a Petition to Legislature. Hartford, 1878.

Two Lectures on Lister's Antiseptic Method of Treating Surgical Injuries. By J. M. Little, M.D. One of the Series of American Lectures, edited by E. C. Seguin, M.D. New York: Putnam & Sons, 1878.

The Diagnosis of Progressive Locomotor Ataxia. By E. C. Seguin, M.D. One of the Series of American Clinical Lectures, edited by E. C. Seguin, M.D.

Conspectus of Organic Materia Medica and Pharmacal Botany, Comprising the Vegetable and Animal Drugs; their Physical Character, Geographical Origin. Classification, Constituents, Doses, Adulterations, etc. Table of the Tests and Solubilities of the Alkaloids appended By L E. Sayre, Ph. G. Cloth, pp. 220; 1879. Philadelphia: D. G Brinton.

Differential Diagnosis: A Manual of the Comparative Semeiology of the More Important Diseases. By F. D. H Hall, M.D. American Edition with Extensive Additions. Philadelphia: D. G. Brinton. 1879.

Diseases of the Abdomen. Comprising those of the Stomach and Other parts of the Alimentary Canal, Œsophagus, Cœcum, Intestines and Peritoneum. By S. O. Habershon. M.D, London. Third Edition, Considerably Enlarged and Revised. Cloth, pp. 706 1878. Philadelphia: Lindsay & Blakiston.

Science and Practice of Surgery. Including Special Chapters by Different Authors. By F. J. Gant, F.R C.S. Second Edition in two Volumes; cloth. 1878. Philadelphia: Lindsay & Blakiston.

Physiology: Preliminary Course of Lectures. By James T. Whittaker, M.A., M.D. Illustrated. Cloth, pp. 288. 1879. Cincinnati: Robert Clarke & Co.

Atlas of Human Anatomy. Illustrating most of the Ordinary Dissections and Many not usually Practiced by the Student, Accompanied by an Explanatory Text. By Richmond John Godlee, M.S., F.R.C.S. Part I. 1878. Paper covers. Philadelphia: Lindsay & Blakiston.

St. Bartholomew's Hospital Reports. Edited by W. S Church, M.D., and Alfred Willett, F.R.C.S. Volume XIV. 1878. London: Smith, Elder & Co.

Transactions of the Medical Society of the State of Pennsylvania at the 28th Annual Session. Held at Pittsburgh, May, 1878. Volume XII.

The Value of Absent Tendon-Reflex as a Diagnostic Sign in Locomotor Ataxia, with an Analysis of Eight Cases. By A. McLane Hamilton. Reprint from *Boston Medical and Surgical Journal.*

Fifty Years Ago. An Address to the Graduating Class of the Medical College of the Pacific for 1878. By H. Gibbons, M.D.

Report of the Quebec Lunatic Asylum for 1876–7.

On a New Modification of the Anterior Splint. By Roswell A. Park, M.D. Reprint from the Transactions of the Illinois State Medical Society, 1878.

Microscopes of the Centennial Exhibition. By J. S. Hunt, M.D.

Sugar Frauds and the Tariff. By H. G. Brown. 1879.

Optic Neuritis with Notes of Three Cases. By C. J. Lundy, M.D. Reprint from *Detroit Lancet*, Dec., 1878.

Brief and Argument for Appellant. N. J. Aiken vs. J. W. Rauch et al.

Annual Report of Pennsylvania Free Dispensary for Skin Diseases.

Artificial Feeding of Infants. By A. Clendinen.

The Testimony of Medical Experts. By W. H. Philips, M.D.

A Case of Acute Puerperal Inversion of the Uterus. By J. Byrne, M.D. Reprint from the *New York Medical Journal*, Oct. and Dec., 1878.

Provision for Insane Criminals. By R. S. Dewey, M.D. Reprint from *Journal of Nervous and Mental Diseases*, Oct., 1878.

Third Bi-Ennial Report of Trustees, Superintendent and Treasurer, of the Illinois Southern Hospital for the Insane, at Anna.

---

THE first number of the first volume of the *St. Louis Courier of Medicine and Collateral Sciences* is at hand — a handsome octavo of 120 pages, with a wood-cut of John Hunter upon the cover.

We have not so far chronicled the appearance of every new mushroom in the field of medical periodical literature, simply because we had better business in hand. We are pleased, however, to make an exception of the journal named above, for two reasons: first, because it is exceptionally good; second, because it is published by the Medical Journal Association of Missouri, an incorporated body of physicians corresponding precisely to the Chicago Medical Press Association. We gladly recognize this publication as own sister of the CHICAGO MEDICAL JOURNAL AND EXAMINER, and wish it long life and prosperity.

### THE METRIC SYSTEM.

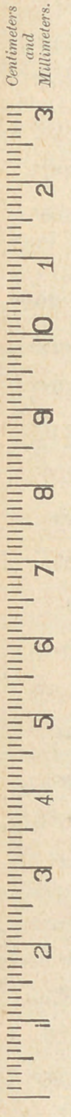

The Metric System and the Centigrade Scale have been exclusively employed in this journal since the beginning of Vol. XXXVII, July, 1878. This system, extensively or exclusively used by many other nations, is legalized in the United States, Great Britain, France, Germany, Russia and Sweden, and has thus become an essential part of the international language of science. It is recommended for adoption in this country by the American Medical Association and other scientific bodies; and is used exclusively by the United States Government in the Marine Hospital Service. To-day no physician can afford to be ignorant of its value, its simplicity and the meaning of its terms.

The subjoined tables and scales, prepared by Prof. W. S. Haines, are regularly reproduced in this journal, for the convenience of readers and correspondents.

### METRIC MEASURES OF LENGTH.

| | | | |
|---|---|---|---|
| Millimeter......... | 0.001 | of a Meter. ......... | 0.03937 in. |
| Centimeter......... | 0.01 | " " .......... | 0.39370 " |
| Decimeter ......... | 0.1 | " " .......... | 3.93707 " |
| **Meter** ........... | **1.** | **Meter**............. | **39.37079** " |
| Decameter......... | 10. | Meters .............. | 393.70790 " |
| Hectometer........ | 100. | " ............. | 3937.07900 " |
| Kilometer .........| 1000. | " ............. | 39370.79000 " |

### METRICAL WEIGHTS.

| | | | |
|---|---|---|---|
| Milligram............ | 0.001 | of a Gram............ | 0.015 gr. |
| Centigram ........... | 0.01 | " " ............ | 0.154 " |
| Decigram ....... .... | 0.1 | " " ............ | 1.543 " |
| **Gram**.............. | **1.** | **Gram**............... | **15.432** " |
| Decagram............ | 10. | Grams................ | 154.323 " |
| Hectogram........... | 100. | " ................ | 1543.234 " |
| Kilogram ............ | 1000. | " ................ | 15432.348 " |

☞ The United States "nickel" five cent piece weighs five grams, and is two centimeters in diameter.

### APPROXIMATE EQUIVALENT OF METRICAL WEIGHTS.

*For Rapid Reference.*

| *Milligrams.* | *Grains.* | *Decigrams.* | *Grains.* |
|---|---|---|---|
| 1 (written 0.001 or \|001)*... | 1/65 | 1 (written 0.1 or \|1)........ | 1 1/2 |
| 2........................ | 1/32 | 2 ........................ | 3 |
| 3........................ | 1/22 | 3 ........................ | 4 1/2 |
| 4 ........................ | 1/16 | 4 ........................ | 6 |
| 5........................ | 1/13 | 5 ........................ | 7 1/2 |
| 6........................ | 1/11 | 6 ........................ | 9 |
| 7........................ | 1/9 | 7 ........................ | 11 |
| 8........................ | 1/8 | 8 ........................ | 12 1/2 |
| 9........................ | 1/7 | 9 ........................ | 14 |

* The decimal *line* instead of points makes errors impossible.

| *Centigrams.* | *Grains.* | *Grams.* | *Grains.* |
|---|---|---|---|
| 1 (written 0.01 or \|.01).. | $^1/_6$ | 1 (written 1. or 1\|).... | 15 |
| 2 .................. | $^1/_3$ | 2 .................. | 30 |
| 3 .................. | $^6/_{13}$ | 3 .................. | 46 |
| 4 .................. | $^7/_{11}$ | 4 .................. | 61 |
| 5 .................. | $^3/_4$ | 5 .................. | 77 |
| 6 .................. | $^9/_{10}$ | 6 .................. | 92 |
| 7 .................. | 1 | 7 .................. | 108 |
| 8 .................. | $1^1/_4$ | 8 .................. | 123 |
| 9 .................. | $1^1/_3$ | 9 .................. | 139 |

A Kilogram=$2^1/_5$ lbs. Avoirdupois.

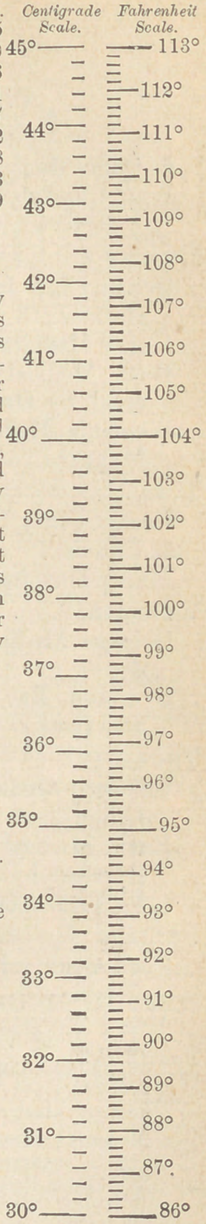

### METRIC FLUID MEASURES.

When using the metric system, fluids are preferably prescribed by weight, employing the gram, its multiples and subdivisions, just the same as with solids, thus avoiding the errors due to refraction, adhesion, and inaccurate measuring vessels. For practical purposes four grams of water may be regarded as equivalent to a fluid drachm of that liquid, and the same may be considered true of tinctures and infusions; syrups, on the average, are about one-third heavier than water, so that a fluid ounce of a syrup will be approximately represented by 43 grams. If preferred, however, fluids may be prescribed by volume in the metric, just as in the present system, using for that purpose the *Cubic Centimeter*, that is, a volume represented by a cube all of whose sides measure one centimeter. An ordinary back-gammon die is usually about this size. One cubic centimeter (written 1 C. C.) = 16.231 minims. It is approximately regarded as one fourth of a fluid drachm.

### APPROXIMATE EQUIVALENTS OF CUBIC CENTIMETER.

| | | | |
|---|---|---|---|
| 0.001 C. C. | = .................. | $^1/_{65}$ | minim. |
| 0.01 " | = .................. | $^1/_6$ | " |
| 0.1 " | = .................. | $1^1/_2$ | " |
| **1. C. C.** | **= ..................** | **15** | **minims.** |
| 4. " | = .................. | 1 | fluid drachm. |
| 16. " | = .................. | 4 | fluid drachms. |
| 32. " | = .................. | 1 | ounce. |

1000 C. C. (usually known as a **Liter**) is a trifle more than one quart, wine measure.

The following prescription—

℞: Potassii bromidi ............℥i.

Elixir aurantii..............fl. ℥viij.

Mix.

would, in metric terms, be written:

| | | |
|---|---|---|
| Potassic bromide....... | 32 | Gm. |
| Orange elixir.......... | 250 | C. C. |

Mix.

Or, in a more finished decimal manner:

| | | |
|---|---|---|
| Potassic bromide....... | 30 | Gm. |
| Orange elixir.......... | 250 | C. C. |

Mix.

### ANNOUNCEMENTS FOR THE MONTH.

### SOCIETY MEETINGS.

Chicago Medical Society—Mondays, Feb. 10 and 24.

West Chicago Medical Society—Mondays, Feb. 3 and 17.

### CLINICS.

MONDAY.

Eye and Ear Infirmary—2 p. m., Ophthalmological, by Prof. Holmes; 3 p. m., Otological, by Prof. Jones.

Mercy Hospital—1:30 p. m., Surgical, by Prof. Andrews.

Rush Medical College—2 p. m., Dermatological and Venereal, by Dr. Hyde; 3 p. m., Medical, by Dr. Bridge.

Woman's Medical College—2 p. m., Dermatological, by Dr. Maynard.

TUESDAY.

Cook County Hospital—2 to 4 p. m., Medical and Surgical Clinics.

Mercy Hospital—1:30 p. m., Medical, by Prof. Hollister.

WEDNESDAY.

Chicago Medical College—1:30 p. m., Eye and Ear, by Prof. Jones.

Eye and Ear Infirmary—2 p. m., Ophthalmological, by Dr. Hotz.

THURSDAY.

Chicago Medical College—1:30 p. m., Medical, by Prof. Quine.

Rush Medical College—3 p. m., Diseases of the Nervous System, by Prof. Lyman; 4 p. m., Diseases of the Chest, by Prof. Ross.

FRIDAY.

Cook County Hospital—2 to 4 p. m., Medical and Surgical Clinics.

Mercy Hospital—1:30 p. m., Medical, by Prof. Davis.

SATURDAY.

Rush Medical College—2 p. m., Surgical, by Prof. Gunn.

Chicago Medical College—2 p. m., Surgical, by Prof. Isham; 3 p. m., Neurological, by Prof. Jewell.

Woman's Medical College—11 a. m., Ophthalmological, by Dr. Montgomery.

Daily Clinics, from 2 to 4 p. m., at the Central Free Dispensary, and at the South Side Dispensary.

